# Sensing Coverage Prediction for Wireless Sensor Networks in Shadowed and Multipath Environment

**DOI:** 10.1155/2013/565419

**Published:** 2013-10-22

**Authors:** Sushil Kumar, D. K. Lobiyal

**Affiliations:** School of Computer & Systems Sciences, Jawaharlal Nehru University, New Delhi 110067, India

## Abstract

Sensing coverage problem in wireless sensor networks is a measure of quality of service (QoS). Coverage refers to how well a sensing field is monitored or tracked by the sensors. Aim of the paper is to have a priori estimate for number of sensors to be deployed in a harsh environment to achieve desired coverage. We have proposed a new sensing channel model that considers combined impact of shadowing fading and multipath effects. A mathematical model for calculating coverage probability in the presence of multipath fading combined with shadowing is derived based on received signal strength (RSS). Further, the coverage probability derivations obtained using Rayleigh fading and lognormal shadowing fading are validated by node deployment using Poisson distribution. A comparative study between our proposed sensing channel model and different existing sensing models for the network coverage has also been presented. Our proposed sensing model is more suitable for realistic environment since it determines the optimum number of sensors required for desirable coverage in fading conditions.

## 1. Introduction

Wireless sensor networks (WSNs) consist of a large number of densely deployed sensor nodes to monitor and track phenomenon or objects of interest in a specified region. Sensor networks pose a number of challenging conceptual and optimization problems such as localization, deployment, and tracking [1–4]. One of the fundamental problems in wireless sensor networks relates to coverage which refers to how well sensors monitor or track the events. In general, coverage can be considered as the measure of quality of service in a sensor network. The coverage requirements may change after a network has been deployed due to changes in the requirements of an application or environmental conditions [5, 6]. 

Sensing coverage problem has been expressed as area coverage and point coverage. Different applications require different degrees of sensing coverage; for example, some applications may require that a location in a region be monitored by only one sensor, while other applications require significantly higher number of sensors for the same [7, 8]. The most general way of defining sensing coverage is to consider the ratio of sensing area to the area of interest, which means the fraction of area that can be covered by the sensor network. The achievable sensing coverage strongly depends on the node deployment planning. However, sensors should be deployed in such a way that it should maximize the coverage and minimize the required number of sensors. Planned node deployment is not always possible in some unstructured sensor network applications like battlefield surveillance, forest monitoring, and so forth. Therefore, in such applications, sensor nodes are randomly deployed to provide the required coverage. However, it may be important to predict the minimum number of sensor nodes that provide an acceptable level of coverage.

Most of the recent research works focus on deterministic sensing models that generally assume uniform sensing ability of a sensor in all directions. This can be represented by a nice regular disk [7, 8]. But, these models are not suitable for realistic environments. Many of the sensor networks concentrate on detecting specific signals such as thermal energy, acoustic signal, seismic signal, radio waves, light waves, or magnetic field, in the preferred sensing area [9]. These signals are affected by environmental factors like noise, interference, reflection of signals, obstructions in the propagation path, and movement of other objects. Sensing signals have extra power loss due to environmental factors besides path loss. This extra power loss results into large deviations in the received signal strength. The deviation in the received signal strength due to obstructions in propagation path is known as shadowing whereas deviation due to reflections is known as multipath fading. The shadowing and multipath fading phenomena adversely affect the quality sensing coverage randomly. Therefore, a probabilistic sensing model accounting for both the shadowing and multipath effects will be more realistic to deal with sensing coverage problem. However, most of the research works in the literature have considered coverage problem with deterministic sensing model or probabilistic sensing model for ideal environment. To the best of our knowledge, no study has focused on the combined impact of large-scale shadowing and multipath fading on the network coverage in wireless sensor networks. 

In this paper, we have proposed a new sensing channel model that considers combined impact of shadowing fading and multipath effects. A mathematical model for calculating coverage probability in the presence multipath fading combined with shadowing is derived based on received signal strength. The sensing coverage problem under shadowing and multipath environment with node deployment following Poisson distribution is analytically explored. A comparative study between the proposed sensing channel model and different existing sensing models, namely, shadow fading sensing model [9], disk sensing model [10], and Elfes sensing model [11] in terms of network coverage has been carried out. Further, the impact of regular and random node deployment on network coverage has also been investigated. 

The rest of the paper is organised as follows.  Section 2 presents the research work related to coverage problem already reported in the literature. In Section 3, the details of proposed sensing channel model along with the assumptions made for modeling are presented. A mathematical model for probabilistic coverage probability is presented in Section 4. The analysis of coverage probability with node deployment following Poisson distribution is presented in Section 5. In Section 6, a model for coverage probability for regular node deployment has been presented. In Section 7 analytical and simulation results for the proposed model are presented and compared with existing models. Finally, the work presented in this paper is concluded in Section 8.

## 2. Related Work

The coverage problem in the sensor networks has received significant attention for past few years from several researchers [7, 8, 12–17]. In all these research works, authors have considered deterministic sensing models in which the sensing area of a sensor node is assumed to be a regular circular disk. However, disk-based sensing models do not properly represent the sensing characteristics of a sensor. Some research efforts have also been made to explore probabilistic coverage with different types of sensing models [11, 18–20]. Most of these models focused on coverage algorithms and issues of estimating number of sensors to be deployed. In general, these models ignored environmental conditions such as obstructions in propagation path, reflection, interference, and noise which are stochastic in nature. 

 In [9], the author has examined sensing coverage under shadowing-fading environment with asymmetric sensing ability of sensors in a randomly deployed wireless sensor network. The coverage probability has been determined for variation in the received signal power using lognormal shadowing fading only. However the author did not consider the fading effects caused by multipath propagation and wave interference occurring in realistic environment. Further, the author has considered constant path loss exponent for different environment. Moreover, the authors in [5, 21, 22] have shown that consideration of constant path loss exponent is not valid for different environment. However, the path loss exponents play an important role in measuring the sensing radius using RSS. Therefore, a model considering variable path loss exponent will be applicable for different environments. 

## 3. Sensing Channel Model

Sensors are applications-oriented sensing devices having widely different sensing characteristics. To represent the sensing characteristics of a sensor, different sensing models based on the specific sensor device and application environment can be constructed. Further, a circular disk model considered in different works does not capture real channel model. Therefore, to develop a sensing channel model for capturing real sensing characteristics, we assumed non-uniform sensing range for a sensor. It is also assumed that *N* sensors uniformly are deployed in a square sensing field of area *A*. All sensors are considered to be homogenous having the same sensing threshold power *δ* (in dB). The sensing threshold is the minimum required strength of the received signal that can be correctly decoded at the sensor. The sensing range of a sensor is determined by the transmit power of a sensing signal, sensing threshold power, and power attenuation along propagation path. The sensing signal power generated by an event is assumed to be *ρ*_*t*_ (in dB). In this work, we have adopted the well-known propagation fading models, that is, log-normal shadowing fading model and multipath fading model, to construct the proposed sensing channel model.

**Figure 1:**
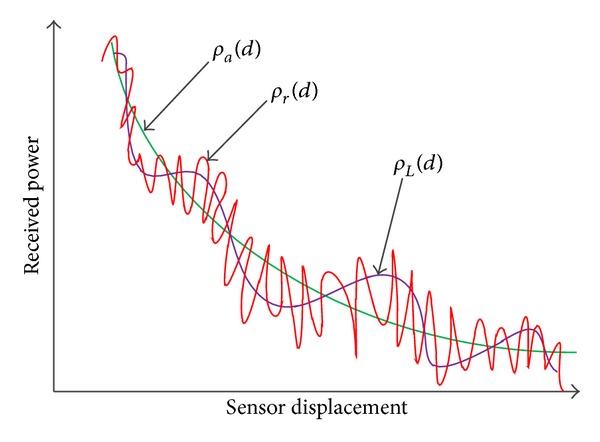
Shadowing and multipath components.

The received signal power *ρ*_*r*_(*d*) (in dB) at a sensor according to log-normal shadowing and multipath fading model can be expressed as [23]

(1) $$\begin{array}{c} \rho_{r}\left(d\right)=\rho_{L}\left(d\right)+\gamma ,\\ \rho_{L}\left(d\right)=\rho_{a}\left(d\right)+\chi_{\sigma },\\ \rho_{a}\left(d\right)=\rho_{t}-\overset{-}{\rho }\left(d_{0}\right)-10\eta \log_{10}\left(\frac{d}{d_{0}}\right), \end{array}$$

where *η* is a path loss exponent that represents the rate in which the path loss increases with distance. $\overset{-}{\rho }\left(d_{0}\right)$ is the mean path loss at reference distance *d*_0_ and *d* is the distance between a sensor and its target. *χ*_*σ*_ is a Gaussian random variable (in dB) with zero mean and variance *σ* ^2^ representing lognormal shadowing effects occurring due to different levels of cutter in the propagation path. The received signal power *ρ*_*L*_(*d*) usually demonstrates a Gaussian distribution with an area mean power *ρ*_*a*_(*d*) (in dB). The parameter *γ* is a random variable (in dB) representing the Rayleigh fading effect caused by multipath propagation and wave interference. The received power *ρ*_*r*_(*d*) is Rayleigh distributed with mean *ρ*_*L*_(*d*) since multipath fading occurs as local fluctuations around the local mean power (cf.  Figure 1). The normalized pdf of *γ* can be expressed as [23]

(2) $$\begin{array}{c} f_{\gamma }\left(y\right)=\frac{y}{{Ω}^{2}}e^{\left[-y^{2}/2{Ω}^{2}\right]}, \end{array}$$

where $Ω=\sqrt{\left(2/\pi \right)}10^{\rho_{L}(d)/20}$ (since *ρ*_*L*_(*d*) = 20log⁡_10_(*m*), *m* is the mean of Rayleigh distribution).

 The sensing threshold power of a sensor is assumed as

(3) $$\begin{array}{c} \delta =\rho_{t}-\overset{-}{\rho }\left(d_{0}\right)-10\eta \log_{10}\left(\frac{R}{d_{0}}\right), \end{array}$$

where *R* is the average sensing radius. The reference distance *d*_0_ in wireless sensor network is generally assumed to be 1 meter, and therefore $\overset{-}{\rho }\left(d_{0}\right)$ becomes a constant. The parameters *η* and *σ* depend on environmental factors. The path loss exponent *η* is very important parameter for coverage and assumed to be constant. However this consideration is not valid for different environments [5, 21, 22]. The value of path loss exponent *η* may differ for line-of-sight (LOS) and Non line-of-sight (NLOS) in the same sensor network. The path loss exponent *η* typically takes value between 2 and 6. The value of *η* can be considered between *η*_min⁡⁡_ (minimum value) and *η*_max⁡_ (maximum value). For the LOS case, when *η* = *η*_min⁡_, the sensing radius *R* attains the maximum value *R*_max⁡_. Therefore, (3) can be represented as

(4) $$\begin{array}{c} \delta =\rho_{t}-\overset{-}{\rho }\left(d_{0}\right)-10\eta_{\min}\log_{10}\left(\frac{R_{\max}}{d_{0}}\right). \end{array}$$

From (3) and (4), the average sensing radius can be expressed as

(5) $$\begin{array}{c} R=d_{0}{\left[\frac{R_{\max}}{d_{0}}\right]}^{\eta_{\min}/\eta }, \end{array}$$

where *η* ∈ [*η*_min⁡_, *η*_max⁡_].

The fading effects depend on the parameters *η*, *σ*, and Ω. Any change in one of these parameters reflects the variation in sensing radius of a sensor. These parameters represent the sensing characteristics of a specific sensor and the application environments and can be used in determining average sensing radius for different environment.

## 4. Random Network Coverage

The network coverage refers to the fraction of area covered by the sensors. It depends on the sensing model used, number of sensors deployed, and the sensor deployment approach followed. In this section, we have described the three existing and widely used sensing models, namely, disk sensing model, Elfes sensing model, and shadow fading sensing model for wireless sensor network [2, 11–13, 16, 17, 20, 24, 25]. These models are further used for comparison and verification of our model. 

Further we have derived coverage probability using our proposed sensing channel model under the effect of Rayleigh fading and lognormal shadowing combined with it. In the next parts of this section, we study the impact of different sensing models on the wireless sensor network to achieve the desired network coverage.

### 4.1. Coverage Using Disk Sensing Model

In the disk sensing model, each sensor has a constant sensing range *r*. The sensing region of a sensor is a disk of an area  *a* = *πr* ^2^. A sensor can only sense the environment and detect the event within its sensing range. A target is said to be covered if it is within the sensing area of a sensor. The probability of target detection by arbitrary sensor is defined as the ratio of sensing area to network area. Therefore, it can be expressed as  *P*_*d*_ = *a*/*A*, where *A* is network area in which *N* sensor nodes are deployed uniformly. The probability (*P*_*c*_) of target detection by at least one of the *N* sensors can be expressed as

(6) $$\begin{array}{c} P_{c}=1-{\left(1-P_{d}\right)}^{N}. \end{array}$$

By applying the equality approximation [1−*x*]^*n*^ ≈ *e* ^−*nx*^ as *n* is very large, (6) can be rewritten as

(7) $$\begin{array}{c} P_{c}=1-\exp\left(-\frac{N\pi r^{2}}{A}\right). \end{array}$$

This formula is used in Boolean sensing model [10]. Here we presented an alternative way to approximate (6) into (7). The Boolean sensing model has been widely used in the study of wireless sensor networks [12, 13, 16, 17]. This model can be used for comparative study with different probabilistic sensing models to determine the required number of sensors for achieving a desired coverage probability.

### 4.2. Coverage Using Elfes Sensing Model

The Elfes sensing model considers the uncertainty in sensing capability of a sensor. The sensing capability is represented by physical parameters of different types of sensor. The Elfes sensing model is widely accepted in the literatures [2, 11, 20] and is used for measuring the performance of different application oriented sensing models. According to Elfes sensing model, the probability that a sensor node sensed an event is between points *R*_min⁡_ and *R*_max⁡_ as given by [2]

(8) Pd=πRmin⁡2A+2πAα2×[(1+αRmin⁡)−e−α(Rmax⁡  −Rmin⁡  )(1+αRmax⁡  )],

where *A* is the area of sensing field and *α* is the physical parameter of a sensor. By substituting *P*_*d*_ in (6), sensing coverage probability can be determined.

### 4.3. Coverage Using Shadowing Fading Sensing Model

The shadowing fading sensing model given in [9] considers the shadowing effects occurring due to obstruction in the propagation path. This model has been widely used in the study of wireless sensor network [2, 24, 25]. The coverage probability for randomly deployed network can be expressed as

(9) $$\begin{array}{c} P_{c}=1-\exp\left(-\frac{N}{A}\overset{R_{\max}}{\underset{0}{\int }}2\pi rQ\left(\frac{10\eta \log_{10}\left(r/R\right)\;}{\sigma }\right)dr\right). \end{array}$$

The coverage probability depends on the shadowing fading parameter *σ*, distance *r* between a sensor and its target to be sensed, and the average sensing radius *R*.

### 4.4. Coverage Using Lognormal Shadowing Fading and Rayleigh Fading

A channel may be subject to both the lognormal shadowing fading and Rayleigh fading effects. The receiver signal power of a sensor varies in all directions since it is received from different propagation paths and suffers from different amount of shadowing and multipath fading loss. Therefore, the sensing radius of a sensor is no longer uniform in all directions. With such assumptions and according to (1), the received sensing power of a sensor at distance *r* from a target can be expressed as

(10) $$\begin{array}{c} \rho_{r}\left(r\right)=\rho_{t}-\overset{-}{\rho }\left(d_{0}\right)-10\eta \log_{10}\left(\frac{r}{d_{0}}\right)+\chi_{\sigma }+\gamma . \end{array}$$

The probability that a sensor S1 detects an event occurring at *r* can be expressed as

(11) Pd(r)=P(ρr(r)>δ)=P(χσ+γ>10ηlog⁡10(rR)),

where *P*(·) denote the probability function. *χ*_*σ*_ is a Gaussian random variable with zero mean and variance *σ* ^2^ and represents shadowing effects in the propagation path. *γ* is a random variable that represents Rayleigh fading to model the multipath effects in propagation path. Therefore, the detection probability *P*_*d*_(*r*) can be expressed as

(12) Pd(r)=∫0∞∫10ηlog⁡10(r/R)−y∞12πσ2e[−(x2/2σ2)]yΩ2e[−y2/2Ω2]dx dy    =∫0∞Q(10ηlog⁡10(r/R)−yσ)yΩ2e[−y2/2Ω2]dy.

By applying one point Gauss-Laguerre formula ∫_0_^*∞*^ *e* ^−*t*^ *f*(*t*)*dt* = *f*(1), the detection probability *P*_*d*(*r*)_ can be written as

(13) $$\begin{array}{c} P_{d}\left(r\right)=Q\left(\frac{10\eta \log_{10}\left(r/R\right)-Ω\sqrt{2}}{\sigma }\right). \end{array}$$

**Figure 2:**
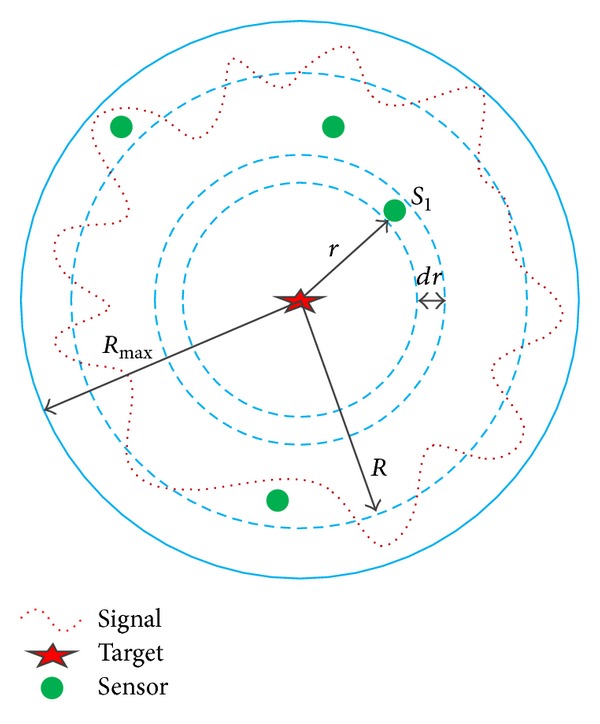
Sensing area for coverage probability analysis.

In Figure 2, the sensor S1 is located at distance *r* from the target located at the origin of the circle. *R*_max⁡_ is the maximum sensing range of a sensor. *R* is an average sensing radius of a sensor. Assume that *r* is continuous and *dr* approaches 0, and the probability that the target is detected by an arbitrary sensor placed in the specified area 2*πr* *dr* of network area *A* can be calculated as follows

(14) $$\begin{array}{c} P_{d}=\frac{1}{A}\overset{R_{\max}}{\underset{r=0}{\int }}P_{d}\left(r\right)\times 2\pi r\;dr. \end{array}$$

Now, the coverage probability can be calculated using Figure 2 as follows. By substituting *P*_*d*_(*r*) into (14), the probability that a target is sensed by an arbitrary sensor node placed in the specified area 2*πr* *dr* of network area *A* can be expressed as

(15) $$\begin{array}{c} P_{d}=\frac{1}{A}\overset{R_{\max}}{\underset{r=0}{\int }}Q\left(\frac{10\eta \log_{10}\left(r/R\right)-Ω\sqrt{2}\;}{\sigma }\right)\times 2\pi r\;dr. \end{array}$$

According to (6), The coverage probability *P*_*c*_ can be expressed as

(16) Pc=1−exp⁡(−NA∫0Rmax⁡2πrQ(10ηlog⁡10(r/R)−Ω2  σ)dr).

This equation can be used in network planning to predict the required number sensors to achieve desired network coverage in a specified environment or to predict the expected coverage probability for a specific number of sensors to be deployed.

## 5. Coverage Probability Prediction Using Poisson Node Distribution

The sensing coverage also depends on how the nodes in the network are distributed. Papers [2, 12, 13] have derived the equation for calculating coverage probability using Poisson node distribution. In this section, we validate the coverage probability obtained in (16) analytically by using Poisson distribution for node deployment. We also derived coverage probability using Poisson node distribution for combined channel effect of multipath fading and shadowing fading. It is assumed that *N* sensor nodes are deployed following Poisson distribution in the network area *A* with node density *µ* equal to *N*/*A*. Let *k* be the number of sensor nodes belonging to a circular band of area 2*πr* *dr*. The probability mass function of *k* using Poisson distribution can be expressed as

(17) $$\begin{array}{c} \emptyset \left(k,µ\right)=\frac{e^{-µ2\pi rdr}{\left(µ2\pi r\;dr\right)}^{k}}{k!},\;k=0,1,2,\ldots . \end{array}$$

The probability that the target cannot be detected by all sensors located in the circular band at distance *r* from the target can be expressed as

(18) Pu(r)=  e−µ2πrdr(µ2πr dr)00![1−Pd(r)]0+e−µ2πrdr(µ2πr dr)11![1−Pd(r)]1+e−µ2πrdr(µ2πr dr)22![1−Pd(r)]2+⋯=e−2µπrdr[1+(µ2πr dr[1−Pd(r)])11!    +(µ2πr dr[1−Pd(r)])22!+⋯]=e−2πµrPd(r)dr.

The probability that the target cannot be detected by any of the sensor in the network area implies that there is no sensor existing in the sensing range of 0 to *R*_max⁡_ and is given by

(19) $$\begin{array}{c} P_{nu}\left(r\right)=\overset{R_{\max}}{\underset{r=0}{\prod }}e^{-2\pi µrP_{d}(r)dr}\;. \end{array}$$

By simplifying the above equation, we get

(20) $$\begin{array}{c} P_{nu}\left(r\right)=e^{-\overset{R_{\max}}{\underset{0}{\int }}2\pi µrP_{d}(r)dr} \end{array}$$

that in other words, *P*_*nu*_(*r*) represents the probability that a target is not detected by any sensor. The probability that the target is sensed by at least one of the *N* sensors or the coverage probability can be expressed as

(21) $$\begin{array}{c} P_{c}=1-e^{-\overset{R_{\max}}{\underset{0}{\int }}2\pi µrP_{d}(r)dr}. \end{array}$$

By substituting *µ*, we get

(22) $$\begin{array}{c} P_{c}=1-e^{-\overset{R_{\max}}{\underset{0}{\int }}(2\pi NrP_{d}(r)/A)dr}. \end{array}$$

Further, sensing coverage probability for combined shadowing fading and Rayleigh fading channel effect can be calculated by substituting *P*_*d*_(*r*) from (13) into (22). Therefore, the coverage probability can be expressed as

(23) Pc=1−exp⁡⁡(−  NA∫0Rmax⁡2πrQ(10ηlog⁡10(r/R)−Ω2  σ)dr).

The coverage probability expressed here is the same as given in (16) for the case of combined shadowing fading and Rayleigh fading. This indicates that the sensing coverage for random node deployment is applicable for high-density wireless sensor network.

## 6. Regular Sensor Node Deployment and Coverage Prediction

**Figure 3:**
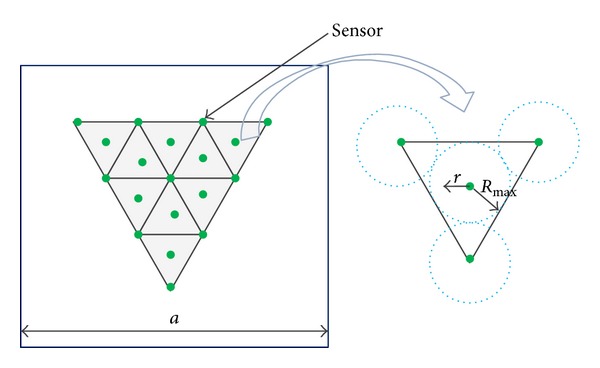
Triangular sensor deployment.

Sensor deployment is an important issue in wireless sensor network, since it impacts the cost of deployment and number of sensors required for desired coverage. Node deployment schemes are broadly classified into two categories—regular deployment and random deployment. In this section, a regular node deployment scheme to determine coverage probability is presented. This scheme will be later used for a comparative study between sensing coverage probability using regular node deployment and random node deployment schemes. In Figure 3, we have considered a sensing field of squared region with side *a*, and the region is partitioned into small equilateral triangular subregions. Further, we assumed that the sensors are placed at the corners and the center of an equilateral triangle. Sensing radius of node varies between 0 and *R*_max⁡_. The maximum sensing radius *R*_max⁡_ is represented by the radius of the incircle of equilateral triangle. Now, the coverage probability can be estimated as the ratio of sensing region inside the triangle to the area of the triangle and represented as

(24) Cf=πr2+πr2/6+πr2/6+πr2/633  Rmax⁡2  =π23  [rRmax⁡]2.

By putting *r* = *R*_max⁡_ in (24), the maximum achievable coverage probability is $\pi /2\sqrt{3}$. Therefore, we can approximate the number of sensor nodes required to cover the sensing field using triangular sensor node deployment as

(25) $$\begin{array}{c} N_{s}=\frac{1}{2\sqrt{3}}{\left[\frac{a}{R_{\max}}\right]}^{2}. \end{array}$$

From the above equation, we can compute that approximately 321 sensor nodes are required to cover a sensing field of side *a* = 500 m and maximum sensing radius *R*_max⁡_ = 15 m. 

## 7. Results and Discussion

In this section, we have presented, the simulation and numerical results to show the impact of sensing channel model under shadowing and multipath fading environments on the network coverage. In the simulation, the entire sensing field is assumed to be a square with area *A* = 1000 × 1000 m^2^. The maximum sensing radius *R*_max⁡_ is assumed to be 20 m. The sensing field is partitioned into small equilateral triangular subregions of side 40, m and a target is located at the center. If the target is sensed by the any one of the sensors, the area of triangle is assumed to be covered by the network. Coverage probability is calculated as the ratio of average number of triangles sensed to the total number of triangles in the sensing field. Others parameters used in simulation are shown in Table 1.

The normalized sensing radius approximated using (5) for different environment is shown in Table 2. While calculating sensing radius, it is assumed that the standard deviation *σ* of lognormal shadowing *χ*_*σ*_ is strongly related to the path loss exponent *η*. For nonshadowing environment, *η* = 2, *σ* = 0, Ω = 0, and the sensing radius attains maximum value, that is, *R*/*R*_max⁡_. The small frequent changes in the path loss exponent can be represented as combined effect of *σ* (shadowing) and Ω (multipath) while keeping the *η* constant for a particular environment. For example, a small variation of 0.05 in the path loss exponent *η* is assumed as 1 dB variation in *σ* and/or Ω. It is clearly noticeable from Table 2 that as *σ* and/or Ω for shadowing and/or multipath environment increases, the average normalized sensing radius significantly decreases. For example, the value of *R*/*R*_max⁡_ reduces from 1 to 0.0811 when *σ* and/or Ω varies from 0 to 3 dB, and for *σ* and/or Ω = 12 dB, the value of *R*/*R*_max⁡_ becomes approximately half of the maximum sensing radius. The increase in the shadowing and multipath parameters *σ*/Ω results in falling off in the sensing coverage. 

**Figure 4:**
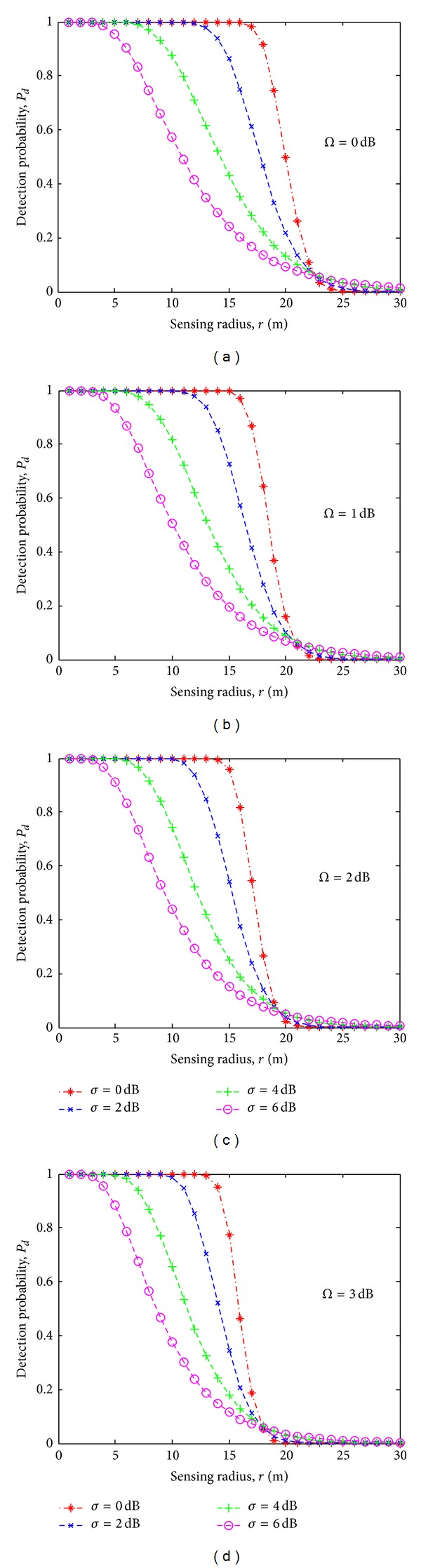
Detection probabilities *P*_*d*_ versus the sensing radius *r* for different shadowing and multipath environments.

Figures 4(a), 4(b), 4(c), and 4(d) illustrate the results of detection probability *P*_*d*_ versus the sensing radius *r* for different shadowing and multipath environment. These results are obtained by applying the derivation given in (13). For the nonshadowing (*σ* = 0) and nonmultipath (Ω = 0) environment, the detection probability at maximum sensing radius is approximately 0.5. In the shadowing and multipath environment, detection probability starts degrading with the decreasing sensing radius beyond 25% of *R*_max⁡_. For example, in Figure 4(c) for Ω = 2 dB and *σ* = 6 dB, the detection probability at the maximum sensing radius is 0.1 which is degraded by 80% of that for a nonshadowing and nonmultipath environment given in Figure 4(a). For Ω = 3 dB and *σ* = 6 dB in Figure 4(d), the detection probability at maximum sensing radius is zero. The higher values of Ω and *σ* indicate that the detection probability degrades drastically with the decreasing sensing radius.

**Figure 5:**
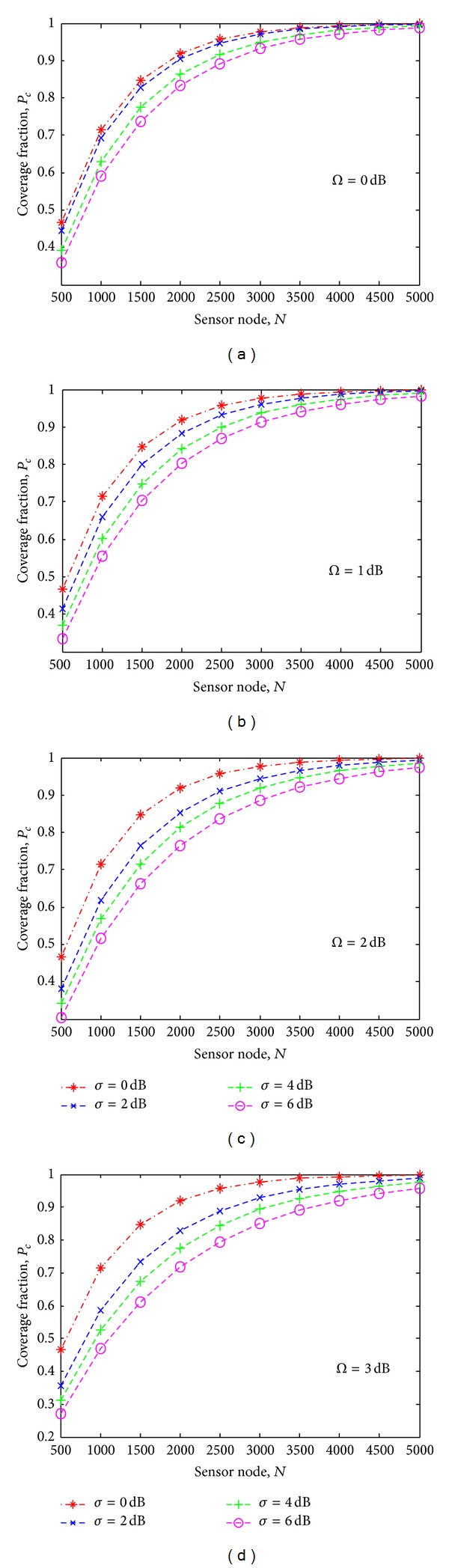
Coverage probability *P*_*c*_ versus number of sensor nodes *N* for different shadowing and multipath environments.

Figures 5(a), 5(b), 5(c) and 5(d) illustrate the results of sensing coverage probability *P*_*c*_ versus number of sensor nodes randomly distributed for different shadowing and multipath environment. These results are obtained by applying the derivation given in (16). From the figure, it is observed that the sensing coverage for nonshadowing *σ* = 0 dB and nonmultipath environment Ω = 0 is approximately 56% of that is obtained in the regular node deployment for the similar environment. However, 91% sensing coverage for Ω = 0 dB and *σ* = 6 dB as shown in Figure 5(a) can be achieved with *N* = 2700 sensor nodes, which is almost 3.7 times the number of sensor nodes required for regular node deployment. For the multipath effects, sensing coverage decreases by approximately 6% of that for nonmultipath and nonshadowing. In addition, to achieve a satisfactory sensing coverage of *P*_*c*_ = 0.90 for different environment (Ω = 1 dB, *σ* = 6 dB), (Ω = 2 dB, *σ* = 6 dB), and (Ω = 3 dB, *σ* = 6 dB), the number of sensor nodes required is approximately *N* = 3000, *N* = 3200, and *N* = 3600, respectively. Therefore, from above results, it is clear that for a harsh shadowing and multipath environment, we need higher number of sensor nodes to achieve the satisfactory coverage.

**Figure 6:**
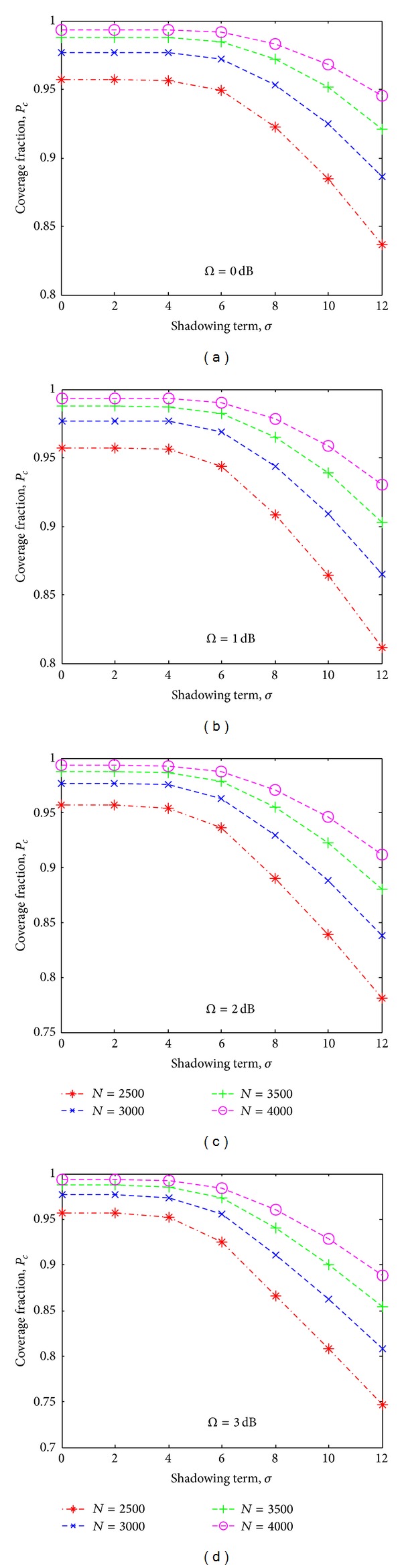
Coverage probability *P*_*c*_ versus shadowing *σ* term and multipath term Ω for different sensor nodes.

Figure 6 illustrates the results of coverage probability *P*_*c*_ versus shadowing term *σ* and multipath term Ω for different number of sensor nodes deployed. These results are obtained by applying the derivation given in (16). It is observed that the sensing coverage decreases sharply with the increase in shadowing and multipath effects. The coverage probability is inversely proportional to *σ* for Ω ≥ 2 dB and *σ* ≥ 4 dB. The harsh shadowing and multipath environment results in decrease of sensing coverage for smaller number of sensor nodes. Further, the sensing coverage for Ω = 3, *σ* = 10 dB, and *N* = 2500 is about 0.80, that is, 84% of that for nonshadowing and nonmultipath environment.

**Figure 7:**
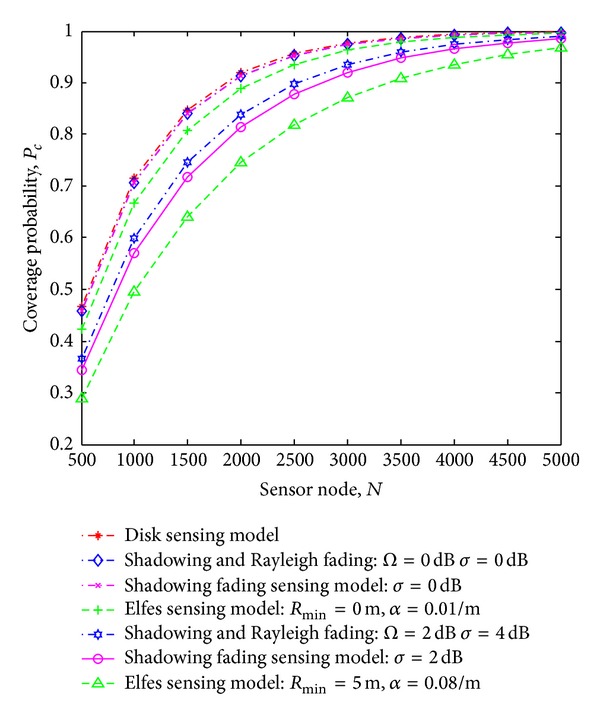
Coverage probability *P*_*c*_ versus Number of sensor nodes using different sensing models.

Figure 7 shows the comparative study of coverage probability for different number of nodes between combined shadowing and Rayleigh fading sensing model and other sensing models. It is observed that the best coverage probability is achieved for Disk sensing model. However, the sensing coverage probability for the proposed combined shadowing and Rayleigh fading sensing model for Ω = 0 dB, *σ* = 0 dB, and shadowing fading model for *σ* = 0 dB are approximately the same as for the disk sensing model. In addition, to achieve a satisfactory sensing coverage, that is, *P*_*c*_ = 0.90, the number of sensor nodes required is *N* = 1800 for combined shadowing and Rayleigh fading model, and nonshadowed environment. While to achieve the satisfactory coverage using Elfes sensing model for *R*_min⁡_ = 0 and *α* = 0.01/m, the number of nodes required is *N* = 2100. From the results, it is also clear that the coverage of a sensor network under the Elfes sensing model requires more nodes than in disk sensing model. The proposed shadowing and Rayleigh fading sensing model provides better network coverage as compared to the Elfes sensing model and shadowing fading sensing model.

**Figure 8:**
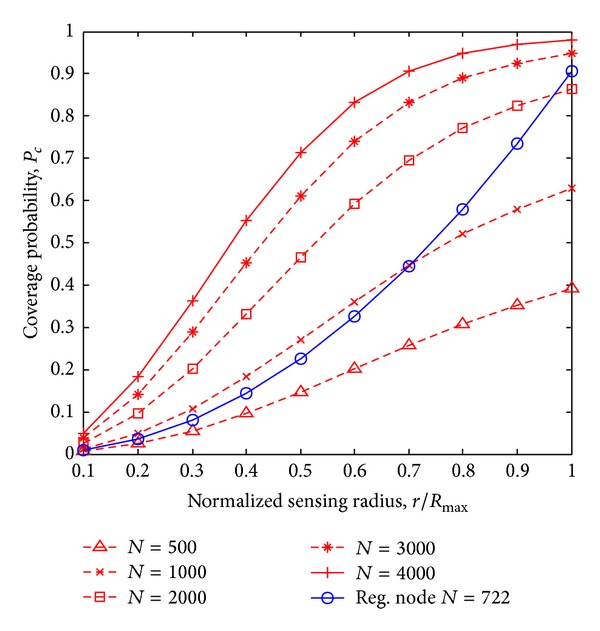
Coverage probability *P*_*c*_ for regular deployment scheme and random deployment scheme.

Figure 8 illustrates the results of coverage probability *P*_*c*_ for normalized sensing radius *r*/*R*_max⁡_ using different number of sensor nodes in both the random and regular node deployment. The coverage probability for varying normalized sensing radius with *N* = 500, *N* = 1000, *N* = 2000, *N* = 3000, and *N* = 4000 is obtained using derivation given in (16). The coverage probability for regular node deployment with *N* = 722 is also obtained using derivation given in (24). It is observed that coverage probability *P*_*c*_ increases with the increase in sensing radius r. The maximum sensing coverage *P*_*c*_ = 0.91 can be achieved for regular node deployment when sensing radius is maximum or *r*/*R*_max⁡_ = 1. For the random node deployment, approximately *N* = 2500 sensor nodes are required to provide the same coverage probability (*P*_*c*_ = 0.91), which is 3.4 times of sensor nodes required for regular node deployment. Though the regular node deployment needs smaller number of nodes for the same coverage, it is not always possible to have this deployment. For example, battlefield, forest regions, hill stations, and oceans require random node deployments due to varying geographic and environmental conditions.

## 8. Conclusion

In this paper, a new sensing channel model which considers combined impact of shadowing fading and multipath effects has been proposed. A mathematical model for calculating detection probability and coverage probability is derived. It has been observed that detection probability as well as sensing coverage degrades with a decrease in average sensing radius in the fading environment. It is also observed that the required number of sensor nodes increases for desired coverage where fading effects are more pronounced. It is evident that the proposed sensing model provides better network coverage for a real environment as compared to other probabilistic sensing models existing in the literature. Therefore, the proposed model will be more useful to evaluate the performance of wireless sensor networks in realistic environment. In the future work, this model could be used to investigate sensing coverage considering interference effects.

## Figures and Tables

**Table 1:** Simulation parameters and their values.

Maximum sensing radius *R*_max⁡_	20 m
Path loss exponent *η*	4
Standard deviation of *χ*_*σ*_ *σ*	0 dB to 12 dB
Multipath component Ω	0 dB to 10 dB
Area of sensing field *A *	1000 × 1000 m^2^
Number of sensor nodes *N *	500 to 5000

**Table 2:** Normalized average sensing radius for different shadowing and multipath environments.

*σ* and/or Ω	0 dB	1 dB	2 dB	3 dB	4 dB	5 dB	6 dB	7 dB	8 dB	9 dB	10 dB	11 dB	12 dB

*R*/*R*_max⁡_	1	0.929	0.867	0.811	0.761	0.716	0.676	0.640	0.606	0.576	0.549	0.524	0.500
